# Total tumor volume as a prognostic value for survival following liver resection in patients with hepatocellular carcinoma. Retrospective cohort study

**DOI:** 10.1016/j.amsu.2020.04.001

**Published:** 2020-04-07

**Authors:** Hazem M. Zakaria, Mahmoud Macshut, Nahla K. Gaballa, Ahmed E. Sherif, Mohammed E. Abdel-Samea, Mohamed Abdel-Samiee, Ibrahim Marwan, Taha Yassein

**Affiliations:** a Department of Hepatopancreatobiliary and Liver Transplant Surgery, National Liver Institute, Menoufia University, Menoufia, Egypt; b Department of Anesthesia and Intensive Care, National Liver Institute, Menoufia University, Menoufia, Egypt; c Department of Diagnostic and Intervention Radiology, National Liver Institute, Menoufia University, Menoufia, Egypt; d Department of Hepatology and Gastroenterology, National Liver Institute, Menoufia University, Menoufia, Egypt

**Keywords:** Hepatocellular carcinoma, Total tumor volume, Milan criteria, Liver resection, Cancer of Liver Italian Program score

## Abstract

**Background:**

Total tumor volume (TTV) can provide a simplified parameter in describing the tumor burden by incorporating the size and number of tumor nodules into one continuous variable. The aim of the study was to evaluate the prognostic value of TTV in resection of hepatocellular carcinoma (HCC).

**Methods:**

Patients who underwent liver resection for HCC between 2012 and 2017 were retrospectively analyzed. Patients were divided into a group with TTV ≤65.5 cm³ (which nearly equal to a single tumor with a diameter of 5 cm), and another group with TTV > 65.5 cm³.

**Results:**

Two hundred and four patients were included in this study (108 patients had TTV ≤ 65.5cm3, and 96 patients had TTV > 65.5 cm³). Ninety patients (44.1%) were within Milan and 114 patients (55.9%) were beyond Milan criteria. Eighteen patients (15.8%) of beyond Milan criteria had TTV ≤ 65.5 cm³, with a median survival of 32 months which is comparable to a median survival of patients with TTV< 65.5 cm³ (38 months, *P* = 0.38). TTV-based Cancer of Liver Italian Program (CLIP) score gained the highest value of likelihood ratio 114.7 and the highest Concordance-index 0.73 among other prognostic scoring and staging systems. In multivariate analysis, independent risk factors for diminished survival were serum AFP level >400 ng/ml, TTV >65.5 cm³, microvascular invasion, postoperative decompensation (all *P* values < 0.05).

**Conclusion:**

TTV is a feasible prognostic measure to describe the tumor burden in patients with HCC. TTV-CLIP score may provide good prognostic value for resection of HCC than other staging systems.

## Highlights

• Total tumor volume (TTV) can provide a simplified parameter in describing the tumor burden.
• This can be done by incorporating the size and number of tumor nodules into one continuous variable.
• TTV, has been shown as a useful prognostic parameter in the prediction of tumor recurrence and survival in liver transplantation.
• However, there is still a scarce clinical series that describe the prognostic value of TTV in liver resection.
• TTV- CLIP score may provide good prognostic value for resection of HCC than other staging systems.

## 1 Introduction

The prognosis of patients with hepatocellular carcinoma (HCC) depends mainly on the functional reserve of the liver and tumor burden that is appraised by the size and number of the tumor nodules. The success of resection depends on the ability to achieve resection with tumor-free margins while leaving behind an adequate liver volume [[1], [2], [3], [4]].

The Milan criteria (single HCC equal or less than 5 cm, or up to 3 nodules no one more than 3 cm) was validated as a selection criteria for patients with HCC candidate for liver transplantation, later on, it was accepted as a prognostic model in liver resection. Some patients who exceeded Milan criteria and underwent curative liver resection were found to have longer survival than expected. This fact has raised the enthusiasm to search for a better efficient prognostic parameter to assess the tumor burden to widen the strict selection criteria and reduce the unnecessary exclusion of some patients in these narrow criteria. So some patients beyond Milan criteria with 2 or 3 HCCs >3 cm, may still have small total tumor volume (TTV) [[5], [6], [7]].

Total tumor volume which collects the number and size of all tumor nodules, has been shown as a useful prognostic parameter in the prediction of tumor recurrence and survival in patients with HCC waiting for liver transplantation. However, there is still a scarce clinical series that describe the prognostic value of TTV in liver resection. The clinical significance of TTV as tumor burden and its impact on long-term patients' outcomes in comparison to other prognostic criteria is still unclear [[8], [9], [10]]. The present study aimed to investigate the prognostic value of TTV in HCC patients who were treated by liver resection.

### 1.1 Patients and methods

We conducted a cohort retrospective study for cirrhotic patients who underwent liver resection for HCC in the period between January 2012 and January 2017 at the National Liver Institute, Menoufia University, Egypt. The study goes ethically in accordance with the World Medical Association Declaration of Helsinki. The research was registered in the Chinese clinical trial registry with a unique identification number ChiCTR2000030403. The work has been reported in line with the Strengthening the Reporting of Cohort Studies in Surgery (STROCSS) criteria [11].

**Fig. 1:**
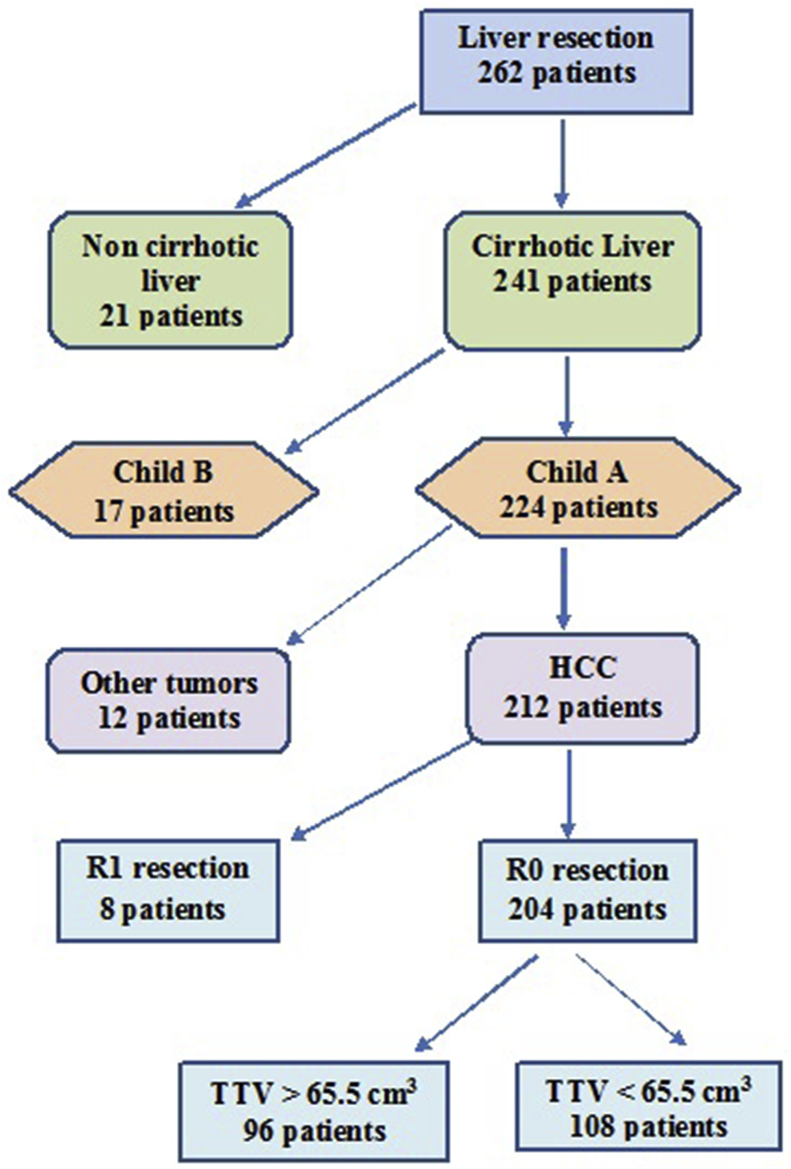
Diagram for selection of the patients with HCC and different TTV.

Patients enrolled in the study had HCC on top of the cirrhotic liver, Child-Turcotte-Pugh (CTP) score A, and underwent R0 curative liver resection (Fig. 1). Preoperative demographics and biochemistry data, operative and postoperative data were collected and analyzed.

Diagnosis of HCC was done preoperatively by its criteria in triphasic computed tomography (CT) scan or magnetic resonance image (MRI) and confirmed histologically after pathological study of the resected specimen. Information about HCC different staging systems was also collected such as tumor-node-metastasis (TNM) staging [12], Barcelona Clinic Liver Cancer (BCLC) staging system [13], Cancer of the Liver Italian Program (CLIP) scoring system [14].

Measurement of the tumor volume was calculated in the first half of patients using this calculation: 4/3 × 3.14 × (maximum radius of the tumor nodule in cm)³ [9,10,15], in the second half of patients the tumor volume was calculated through manual volumetry in multimodality CT scan software which is more accurate than the calculation as some tumors may not be spherical. In the case of multiple tumors, the TTV was calculated through the sum of each tumor nodule. Patients were divided into 2 groups; a group with TTV ≤65.5 cm³ (which nearly equal to a single tumor with a diameter of 5 cm), and another group with TTV > 65.5 cm³.

The TTV was incorporated in the CLIP system for accurate prediction of the outcome of patients with HCC (Table 1). A TTV of 200 cm³ is nearly equivalent to a single tumor nodule with a 7.3 cm tumor diameter or three nodules the diameter of each of it equals nearly 5 cm.

**Table 1:** The original CLIP vs reconstruction of the TTV-based CLIP score.

Parameters	Original CLIP	TTV-based CLIP
**Tumor morphology**		
Single and <50% liver span	0	–
Multiple and <50% liver span	1	–
≥50% liver span	2	–
**Total tumor volume**		
<65.5 cm³	–	0
65.5–200 cm³	–	1
200–500 cm³	–	2
>500 cm³	–	3
**Serum AFP level (ng/mL)**		
<400	0	0
≥400	1	1
**Macrovascular invasion**		
No	0	0
Yes	1	1
**CTP score**		
A	0	0
B	1	1
C	2	2

CLIP: Cancer of the Liver Italian Program, AFP: alpha-fetoprotein, TTV: total tumor volume, CTP: Child-Turcotte-Pugh.

Follow up of the patients was done from the date of surgery up to January 2020 with a median follow up 41 months. Follow up was done by laboratory investigations including alpha-fetoprotein (AFP) and abdominal ultrasound (US) every 3 months in the first 2 years after surgery then every 6 months. For detection of HCC recurrence contrast-enhanced CT scan or MRI was done every 6 months in the first year then yearly.

Early postoperative medical or surgical complications were recorded and classified according to the Clavien Dindo grades of postoperative complications [16]. Post-hepatectomy liver failure (PHLF) or decompensation and its grades have been defined by the International Study Group of Liver Surgery (ISGLS) to describe the increase of INR and serum bilirubin on or after postoperative day 5 [17].

### 1.2 Statistical analysis

Statistical analysis was done using STATA 13 (STATA corp., TX, USA) and SPSS 23 (SPSS Inc., Chicago, IL). Chi-square or Fisher's exact test was used for categorical variables comparison. In continuous variables, the Mann-Whitney *U* test or Kruskal-Wallis H test was used. Overall survival (OS) rates were applied by the Kaplan-Meier method, while differences in survival rates were appraised by the Log-rank test. Cox's proportional hazard model was used for multivariate analysis, the χ2 value of the likelihood ratio test that is related to Cox's proportional model was used to evaluate the categories of each system. Evaluation of different survival data was determined by using Harrell's concordance index (C-index) [18]. The tumor staging or scoring system with a higher value of the χ2 likelihood ratio test and C-index was considered to have superior prognostic power. *P* < 0.05 was considered to be statistically significant.

## 2 Results

### 2.1 Patient characteristics

**Fig. 2:**
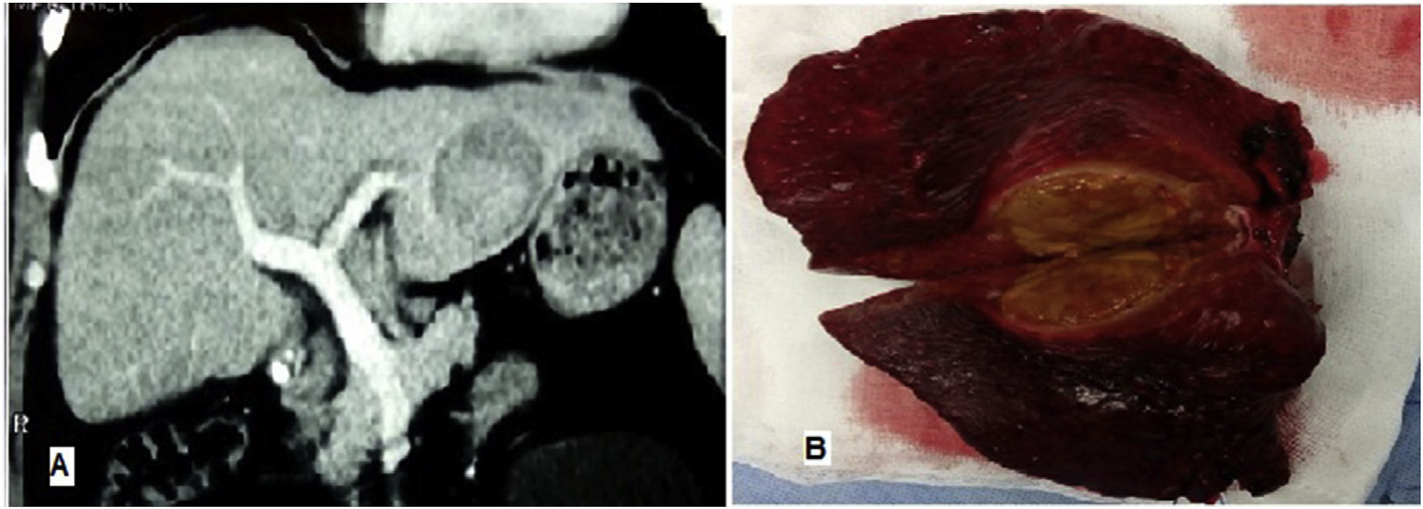
(a) Portal phase triphasic CT scan showing an HCC lesion in the left lateral segment. (B) The specimen post left lateral resection including the HCC lesion size: 5.2 × 4.8 cm & TTV = 65.7 cm^3^.

**Fig. 3:**
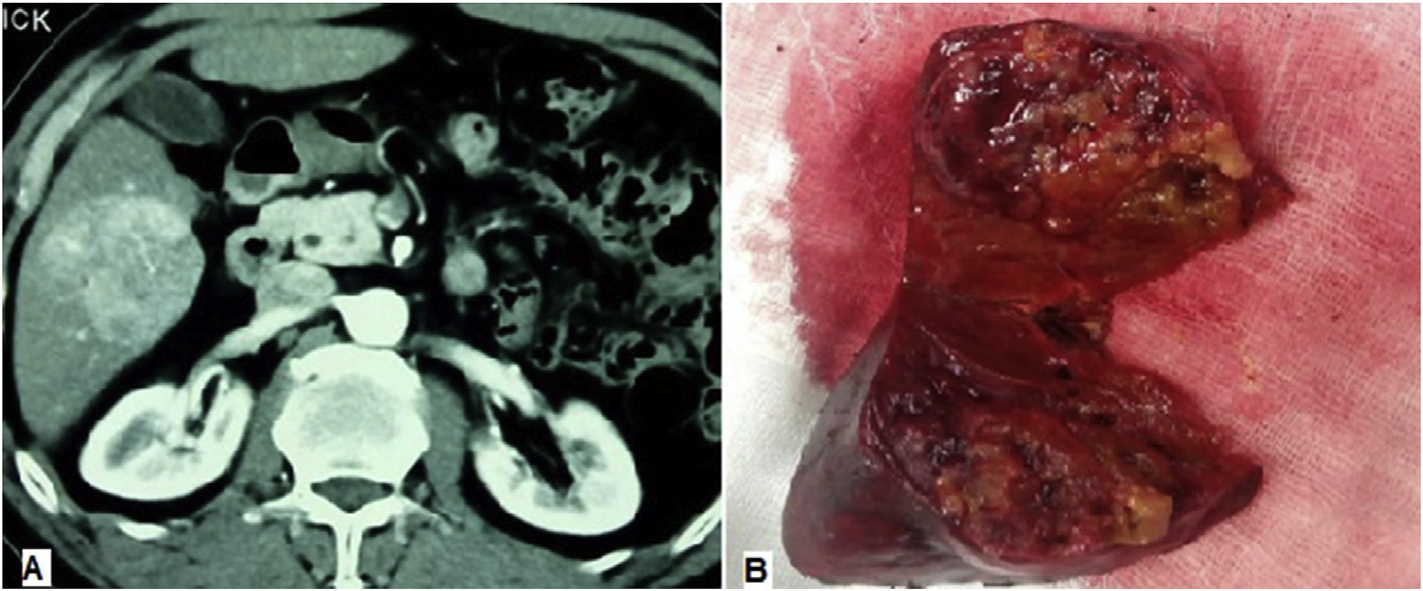
(a) Portal phase triphasic CT scan showing segment VI lesion. (B) The specimen post non anatomical resection size: 5.5 × 5.3 cm & TTV=71 cm^3^.

**Fig. 4:**
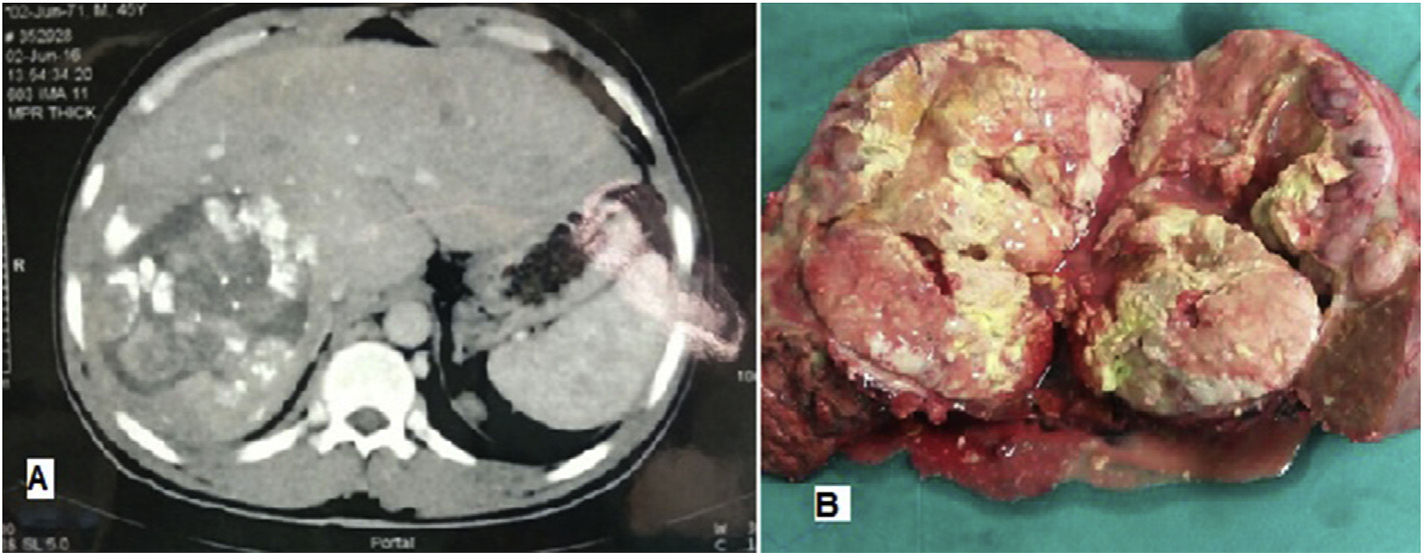
a) Portal phase triphasic CT scan showing right lobe large lesion with lipidol retention post trans arterial chemo-embolization (TACE). (B) The specimen post formal right hepatectomy for HCC with areas of necrosis post TACE, size: 11.9 × 9.6 cm & TTV = 800cm^3^.

Two hundred and four patients were included in this study (108 patients had TTV ≤ 65.5 cm³, and 96 patients had TTV > 65.5 cm³), (Fig. 2, Fig. 3, Fig. 4). The mean age of overall patients was 59 years, and 80.9% of them were male. The most common cause of chronic liver disease was hepatitis C virus (HCV) (91.2%). Solitary tumor was detected in 175 patients (85.8%).

Table 1, shows the (TTV-CLIP) scoring after the replacement of tumor morphology by the TTV as a more accurate measure for tumor burden.

The clinicopathological character and different prognostic staging of the two groups of patients were summarized in Table 2. The operative and postoperative data are in Table 3. In the univariate analysis, there was a significant difference between both groups in serum AFP level (*P* = 0.02), tumor diameter (*P* = 0.01), Milan criteria (*P* = 0.001), operative blood loss (*P* = 0.03), operative time (*P* = 0.04), hospital stay (*P* = 0.04).

**Table 2:** Preoperative clinicopathological data and different staging systems.

Variable	TTV ≤65.5 cm^3^ (n = 108)	TTV > 65.5 cm^3^ (n = 96)	*P*-value
Age			0.69
*Mean* ± *SD*	57.8 ± 4.5	60.2 ± 5.3	
*Range*	47–73	45–71	
Gender			0.86
*Male*	89 (82.4%)	76 (79.2%)	
*Female*	19 (17.6%)	20 (20.8%)	
Etiology of liver disease			1.0
HCV	99 (91.7%)	87 (90.6%)	
HBV	6 (5.6%)	4 (4.2%)	
HCV&HBV	1 (0.9%)	2 (2.1%)	
Others	2 (1.8%)	3 (3.1%)	
Total bilirubin (mg/dl)			0.34
*Mean* ± *SD*	1 ± 0.39	0.81 ± 0.63	
*Range*	0.48–1.9	0.38–1.8	
Albumin (g/dl)			0.75
*Mean* ± *SD*	3.6 ± 0.5	3.78 ± 0.56	
*Range*	2.8–4.5	2.8–4.8	
INR			0.32
*Mean* ± *SD*	1 ± 0.3	1.1 ± 0.22	
*Range*	0.9–1.6	1.0–1.5	
ALT (IU/L)			0.70
*Mean* ± *SD*	51 ± 32	54 ± 39	
*Range*	32–109	9–136	
MELD			0.12
≤9	76 (70.4%)	56 (58.3%)	
>9	32 (29.6%)	40 (41.7%)	
AFP (ng/ml)			**0.02**
*Mean* ± *SD*	290 ± 371	504 ± 1022	
*Range*	2.5–1250	3–11053	
Tumor number			0.22
*1*	88 (81.5%)	87 (90.6%)	
*2*–3	20 (18.5%)	9 (9.4%)	
Tumor Site			0.35
*Right lobe*	59 (54.6%)	61 (63.5%)	
*Left Lobe*	33 (30.6%)	29 (30.2%)	
*Bilobar*	16 (14.8%)	6 (6.3%)	
Tumor diameter (cm)			**0.01**
*Mean* ± *SD*	3.6 ± 0.7	5.8 ± 2.4	
*Range*	3–4.8	5–11	
Macrovascular invasion			0.82
*Absent*	107 (99.1%)	94 (97.9%)	
*Present*	1 (0.9%)	2 (2.1%)	
TNM Stage			0.40
*I*	88 (81.5%)	85 (88.5%)	
*II*	20 (18.5%)	2 (2.1%)	
*III*	0 (0%)	9 (9.4%)	
BCLC			0.16
*0*	10 (9.3%)	0	
*A*	80 (74%)	81 (84.4%)	
*B*	18 (16.7%)	15 (15.6%)	
CLIP			0.4
*0*	73 (67.6%)	63 (65.6%)	
*1*	23 (21.3%)0	24 (25%)	
*2*	12 (11.1%)	7 (7.3%)	
*3*	0	2 (2.1%)	
*4*	0	0	
TTV-CLIP			**0.001**
*0*	75 (69.4%)	0	
*1*	32 (29.6%)	57 (59.4%)	
*2*	1 (0.9%)	20 (20.8%)	
*3*	0	15 (15.6%)	
*4*	0	4 (4.2%)	
Milan Criteria			**0.001**
*Within*	90 (83.3%)	0 (0%)	
*Beyond*	18 (16.7%)	96 (100%)	

SD (standard deviation), ALT (alanine aminotransferase), INR (international normalized ratio), MELD (model of end stage liver disease), CLIP Cancer of the Liver Italian Program, AFP (alpha-fetoprotein), TTV (total tumor volume).

**Table 3:** Operative and postoperative data.

Variable	TTV ≤65.5 cm^3^ (n = 108)	TTV > 65.5 cm^3^ (n = 96)	P value
Type of operation			0.31
*Laparoscopic*	14 (11.1%)	6 (6.2%)	
*Open*	94 (88.9%)	90 (93.8%)	
Type of resection			0.30
*Anatomical*	26 (24.1%)	29 (30.2%)	
*Non anatomical*	82 (75.9%)	67 (69.8%)	
Blood loss (ml)			**0.03**
*Mean* ± *SD*	290 ± 370	588 ± 294	
*Range*	50–1500	100–3500	
Intraoperative blood transfusion (unit)			0.09
*Mean* ± *SD*	1 ± 1	2 ± 3	
*Range*	0–6	0–12	
Intraoperative plasma transfusion (unit)			0.38
*Mean* ± *SD*	2 ± 2	2 ± 4	
*Range*	0–8	0–14	
Operative time (min)			**0.04**
*Mean* ± *SD*	174 ± 40	210 ± 65	
*Range*	168–290	180–420	
Tumor differentiation			0.36
well	14 (13%)	10 (10.4%)	
moderate	75 (69.4%)	60 (62.5%)	
poor	19 (17.6%)	26 (27.1%)	
Microvascular invasion			0.07
*Yes*	19 (17.6%)	29 (30.2%)	
*No*	89 (82.4%)	67 (69.8%)	
Liver decompensation			0.33
*Yes*	34 (31.5%)	38 (39.6%)	
*No*	74 (68.5%)	58 (60.4%)	
Recurrence			0.06
*Yes*	20 (18.5%)	32 (33.3%)	
*No*	88 (81.5%)	64 (66.7%)	
Hospital stay (days)			**0.04**
*Mean* ± *SD*	6 ± 5	9 ± 8	
*Range*	3–15	4–32	
Clavien Dindo grades of complications			0.23
0	69 (63.9%)	46 (47.9%)	
I	16 (14.8%)	19 (19.8%)	
II	12 (11.1%)	14 (14.6%)	
III	6 (5.6%)	9 (9.4%)	
IV	2 (1.9%)	4 (4.2%)	
V	3 (2.8%)	4 (4.2%)	

SD (standard deviation), TTV (total tumor volume).

### 2.2 Comparison of survival distribution in patients within and beyond Milan, both groups of TTV and other different staging systems

Of the studied patients, 90 patients (44.1%) were within Milan and 114 patients (55.9%) were beyond the Milan criteria. The median survival in both groups was 36.5 months and 25 months respectively (*P* = 0.09). Of the patients that exceeded the Milan criteria 18 patients (15.8%) with multiple tumors had TTV ≤ 65.5 cm³ (Table 2), and median survival was 32 months which is comparable to the median survival of patients with TTV< 65.5 cm³ (38 months, P = 0.38).

In patients within Milan criteria the 1, 3, and 5y survival rates were 92.6%, 76.1%, 65.7% respectively, while in patients beyond Milan criteria survival rates were 89.5%, 69.1%, 48.6 respectively. Log-rank: (*P* = 0.09) with no significant difference in survival.

**Fig. 5:**
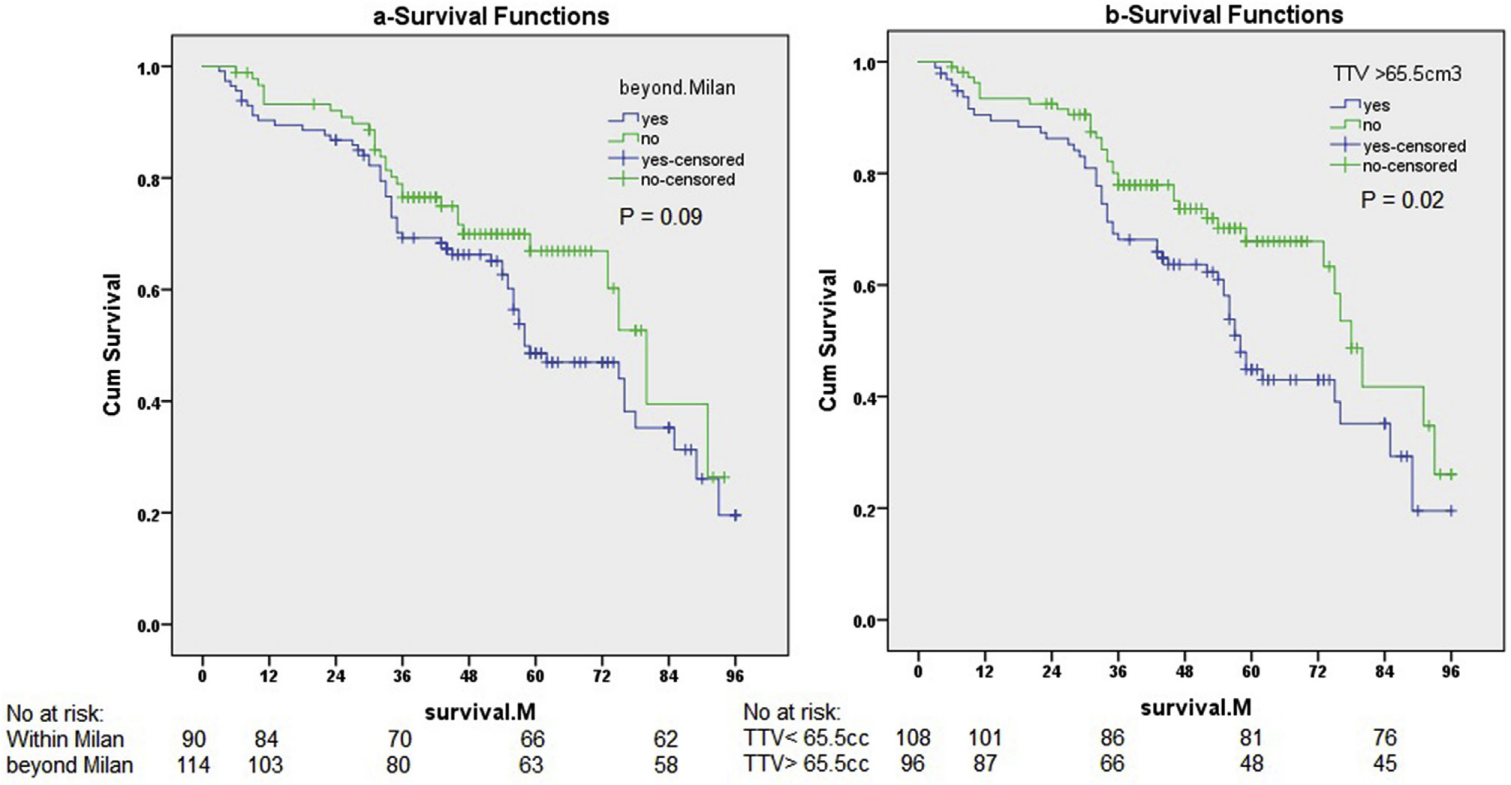
Kaplan-Meier curve (a) for patients within and beyond Milan criteria and (b) patients with TTV≤ 65 cm^3^ and TTV>65 cm^3^.

The 1, 3, and 5y survival rates in patients with TTV≤65 cm³ were 93.2%, 77.2%, 68.4% respectively, while in patients with TTV >65 survival rates were 89.2%, 68.1%, 43.5% respectively. Log rank: (*P* = 0.02), with significant difference in 5y survival (Fig. 5).

The median overall survival times across the TTV-CLIP scores 0–4 were 51.5, 37, 22.5, 18, 10.5 months, respectively with a statistical significant difference in the pair-wise comparison (*P* = 0.02). When the prognostic performance of the TTV-CLIP score was compared to the CLIP score, TNM staging system, BCLC staging system, and Milan criteria, TTV-CLIP gained the highest value of likelihood ratio test as 114.7 and the highest C-index as 0.73 among other prognostic scoring systems (Table 4).

**Table 4:** Comparison of predictive power for survival of different tumor prognostic models in patients with hepatocellular carcinoma.

Variable	Likelihood ratio test	C-index
TTV-CLIP(0,1,2,3,4,5,6)	114.7	0.73
TTV (≤65, >65) cm^3^	103.4	0.64
CLIP(0,1,2,3,4)	98.1	0.56
BCLC (|0,A,B,C)	86.6	0.53
Milan (within, beyond)	100.2	0.61
TNM(0,I,II,III,IV)	72.8	5.1

(Higher likelihood ratio test and C-index were associated with better performance of the stage or score system)TTV (total tumor volume), CLIP (Cancer of the Liver Italian Program), BCLC Barcelona Clinic Liver Cancer staging system, TNM (tumor-node-metastasis) staging.

### 2.3 Univariate and multivariate analysis for risk factors for survival

In univariate analysis (Table 5) the risk factors for 5y survival in all patients with HCC were preoperative total bilirubin level >1.5 mg/dl (*P* = 0.03), serum AFP level >400 ng/ml (*P* = 0.02), macrovascular invasion (*P* = 0.02), TTV > 65.5 cm^3^ (*P* = 0.02), pathological tumor grades III&IV (*P* = 0.4), microvascular invasion (*P* = 0.01), postoperative hepatic decompensation (*P* = 0.01).

**Table 5:** Risk factors for survival in patients with HCC.

Variable	Univariate analysis				Multivariate analysis		
	Number of deaths per cases observed (%)	HR	95%CI	*P* value	HR	95%CI	P value
Age (years)		0.62	1.07–1.28	0.19			
*≥ 60*	31/72 (43.1%)						
< *60*	44/132 (33.3%)						
Gender		0.94	2.13–2.57	0.24			
*Male*	58/165 (35.2%)						
*Female*	17/39 (43.6%)						
Total bilirubin *(mg/dl)*		1.25	0.68–1.49	**0.03**	0.68	0.49–1.12	0.07
≤ *1.5*	36/130 (27.7%)						
> *1.5*	39/74 (52.7%)						
Albumin (gm/dl)		0.81	0.90–1.72	0.25			
*≥ 3.5*	38/117 (32.5%)						
< *3.5*	37/87 (42.5%)						
AFP (ng/ml)		0.72	0.43–0.94	**0.02**	2.07	2.03–3.36	**0.039**
≤ *400*	39/139 (28%)						
> *400*	36/65 (55.4%)						
Portal Hypertension		0.84	0.69–1.06	0.24			
Yes	48/116 (41.4%)						
No	27/88 (30.7%)						
Tumor number		0.96	1.92–2.36	0.16			
single	62/175 (35.4%)						
multiple	13/29 (44.8%)						
TTV (cm^3^)		0.92	0.27–.0.81	**0.02**	2.16	2.08–3.12	**0.05**
≤ *65*	26/108 (24.1%)						
>65	49/96 (51%)						
Macrovascular invasion		1.42	0.46–1.46	**0.02**	1.47	1.03–1.69	0.07
*Absent*	73/201 (36.3%)						
*Present*	2/3 (66.7%)						
Milan criteria		1.07	0.91–1.96	0.09			
*Within*	25/90 (27.8%)						
*Beyond*	50/114 (43.9%)						
Type of operation		1.24	0.68–0.98	0.08			
*Laparoscopic*	5/20 (25%)						
*Open*	70/184 (38%)						
Type of resection		0.84	0.65–1.09	0.42			
*Anatomical*	22/55 (40%)						
*Non anatomical*	53/149 (35.6%)						
Operative time (hours)		0.51	1.25–2.13	0.07			
≤ *3*	15/56 (26.8%)						
> *3*	60/148 (40.5%)						
Grading		1.04	0.91–2.24	**0.04**	0.89	0.63–1.05	0.09
*I,II*	26/91 (28.6%)						
*III*,IV	49/103 (47.6%)						
Microvascular invasion		1.15	1.36–2.09	**0.01**	1.02	1.14–1.83	**0.012**
*Yes*	31/48 (64.6%)						
*No*	45/156 (28.8%)						
Postoperative liver decompensation		0.87	2.17–2.62	**0.01**	1.68	1.56–2.93	**0.041**
*Yes*	40/72 (55.6%)						
*No*	35/132 (26.5%)						

TTV: total tumor volume, AFP: alpha-fetoprotein.

In multivariate analysis Independent risk factors for survival were serum AFP level >400 ng/ml (HR = 2.07, CI = 2.03–3.36, P = 0.039), TTV > 65.5 cm^3^ (HR = 2.16, CI = 2.08–3.12, *P* = 0.05), microvascular invasion (HR = 1.02, CI = 1.14–1.83, *P* = 0.012), postoperative decompensation (HR = 1.68, CI = 1.56–2.93, P = 0.041) (Table 5).

## 3 Discussion

Different scoring and staging systems have been validated as a predictor of long term outcome of patients with HCC, but few studies have evaluated the prognostic efficacy of TTV in patients receiving liver resection [[12], [13], [14],^19^].

TTV can provide a simplified parameter in describing the tumor burden by incorporating the size and number of tumor nodules into one continuous variable, so by analyzing a single parameter may be easier and simpler than analyzing the number and size of tumor nodules separately. It has been proven to be a useful parameter in describing tumor progress mainly in patients with HCC waiting for liver transplant [9,20].

Most of the series identified that larger TTV had more tumor burden with associated larger tumor size and number, high AFP level, macrovascular invasion, and advanced tumor stages with the consequent shorter OS than others with smaller TTV [[21], [22], [23]], as seen also in our study.

Different studies showed that large TTV can predispose to have AFP >400 ng/ml, AFP level has been linked with the aggressive behavior of the tumor cells and disease progression. Also, larger tumors were assumed to have a higher incidence of satellite nodules and vascular invasion. So the consequent relation between larger TTV and aggressive clinicopathological character of HCC led to the valuable studies of the prognostic value of TTV [[21], [22], [23], [24]]. In our study patients with TTV >65.5 cm^3^ had a higher level of AFP and more incidence of microvascular invasion than smaller ones.

Although the Milan criteria are still used as the standard selection criteria for curative treatment in patients with early-stage HCC, patients beyond the criteria may also have small TTV. Lee et al., in their series there were 50 patients beyond Milan criteria and 10% of them presented with ≥4 nodules, but they had relatively small TTV of about 9.4 cm and this is provided a possible explanation that patients beyond Milan criteria had a relatively favorable long-term outcome near to patients within Milan criteria as reported in previous studies [[24], [25], [26]]. It was seen also in our study that there was no significant difference in survival between patients within and beyond Milan criteria.

In one series it was noted that patients with multiple HCC but of moderate sizes had a better outcome than patients with only one tumor but of large size [23].

Li et al., identified that 10.9% of patients were beyond Milan criteria but with TTV less than 73 cm³ [15], and Lee et al., also reported 6.5% patients exceeding the Milan criteria had TTV <65.5 cm³ with good outcome after resection like small TTV [24]. In our series, there was 15.8% patient beyond Milan criteria and had small TTV <65.5 cm³ with comparable outcome.

Li et al. showed a comparable survival after resection of HCC between patients within and beyond Milan criteria. But, there was a significant difference in OS between patients with TTV >73.5 cm3 and patients with TTV≤ 73.5 cm3 [15]. As seen in our study with a distinct difference in OS between patients with TTV >65.5 cm^3^ and patients with TTV≤ 65.5 cm^3^. In the former study, they suggested that TTV between 17.1 cm^3^ and 73.5 cm^3^ can be used as an expanded selecting criteria before resection of HCC [15].

CLIP score was validated in a lot of studies for HCC prognosis [14,27], but one of its variables is relatively subjective as no specific size can be determined, so its reliability to detect the outcome may be compromised. Some studies replaced the tumor morphology section by different values of TTV as modified CLIP score which gained a better prognostic ability than the original one and other staging systems like BCLC staging, Milan criteria and TNM staging [8,15], as seen also in our study that modified TTV-CLIP score has significant difference and more prognostic value than other staging systems for HCC. In one study, the median OS in the TTV-CLIP scores 0–6 were 65.5, 50, 41, 32, 20.5, and 15 months, respectively [15]. In our study, the median survival in TTV-CLIP scores 0–4 were 51.5, 37, 22.5, 18, 10.5 months, respectively with a statistically significant difference.

In one series the independent prognostic predictors for survival were serum sodium level, bilirubin, AFP level and TTV [24]. In other studies, the prognostic determinants were tumor burden and the functional reserve of the liver [15,28]. In our study, TTV, serum AFP, microvascular invasion and postoperative decompensation were independent risk factors for survival.

The possible limitations of this study may be that hepatitis C virus (HCV) was the commonest cause of chronic liver disease and HCC in our study which may be different from other countries where alcoholism, hepatitis B virus (HBV), or non-alcoholic steatohepatitis are the predominant causes, so the tumor character and biology can be different. The TTV calculation in most of our patients was depended on the assumption that the tumor nodules are spherical, but some tumors may be infiltrative or irregular in shape that can result in an inaccurate measure of TTV. The prognostic value of TTV and TTV-CLIP scores still in need of more studies for better validation.

Conclusions: TTV is a good parameter to describe the tumor burden in HCC. Patients with TTV ≤65.5 cm3 can gain better outcomes after resection than patients with larger tumor volumes so it can be used as a selection criteria before resection of HCC. Modified TTV-CLIP score may provide a better prognostic value before resection of HCC than other staging and scoring systems but still need further research.

## Ethical approval

The research was conducted ethically in accordance with the World Medical Association Declaration of Helsinki. The study protocol was approved by the National Liver Institute committee and review board, Menoufia University, Egypt .

## Sources of funding

No funding.

## Author contribution

Hazem Zakaria, Mahmoud Macshut, Nahal Gaballa, Ahmed E Sherif, Mohammad E Abdel-Samea, Mohamed Abdel-Samiee, Ibrahim Marwan, Taha Yassein, actively participated in the preparation, study design, collection of the data and editing of the manuscript. Statistical analysis was done by Hazem Zakaria.

## Trial registry number

1. Name of the registry: Chinese Clinical Trial Registry
2. Unique Identifying number or registration ID: ChiCTR2000030403
3. Hyperlink to the registration (must be publicly accessible):http://www.chictr.org.cn/showprojen.aspx?proj=50331

## Guarantor

Hazem Mohamed Zakaria, Department of Hepatopancreatobiliary & liver transplant surgery, National Liver Institute, Menoufia University, 32511 Shebin El-koom, Menoufia, Egypt. E-mail: hazemlasheenn@yahoo.com Tel: +2 01019353448, +9665071052192 Fax: +20482234586; Tel.: +20482222740.

## Provenance and peer review

Not commissioned externally peer reviewed.

## Consent

The research was conducted ethically in accordance with the World Medical Association Declaration of Helsinki. The patients have given their written informed consent on admission and pre-operative to use their prospective data base and files for research work (and as it is a retrospective study on the previous patients data and records so no need for new consents).

## Declaration of competing interest

No conflict of interest.
